# Effect of direct-acting antivirals on corrected QT interval and cardiac functions in patients with chronic hepatitis C virus infection

**DOI:** 10.1186/s43044-020-0042-y

**Published:** 2020-02-07

**Authors:** Mohamed Gamal Ibrahim, Ahmed Abdelrahman Sharafeldin, Nevine Ibrahim Mousa, Tarek Khairy Mousa, Ahmed Mohamed El Missiri

**Affiliations:** 1 Cardiology Department, Ain Shams University, Abbassia square, Abbasia, Cairo, 11566 Egypt; 2 Department of Internal Medicine, Gastroenterology and Hepatology, Ain Shams University, Abbassia square, Abbasia, Cairo, 11566 Egypt

**Keywords:** Echocardiography, Hepatitis C virus, Direct-acting antiviral, Cardiac function

## Abstract

**Background:**

Hepatitis C virus (HCV) infection is a major public health problem in Egypt. The use of direct-acting antivirals (DAAs) in such patients has been shown to be highly effective. The cardiac safety of such antivirals remains uncertain. This study aimed to assess the effect of the novel DAAs on corrected QT (QTc) interval and on cardiac function using trans-thoracic echocardiography.

**Results:**

This was a prospective cohort study performed on 100 patients suffering from chronic HCV infection. Patients were into two equal groups according to the presence of liver cirrhosis. The group without liver cirrhosis received a daily combination of sofosbuvir 400 mg and daclatasvir 60 mg for 12 weeks while that with liver cirrhosis (Child-Pugh score A or B) received a daily combination of sofosbuvir 400 mg, daclatasvir 60 mg, and ribavirin 600 mg for 12 weeks. Surface ECG and trans-thoracic echocardiography were performed prior to the start of treatment and after 12 weeks of treatment.

At the end of treatment, no changes were observed in QTc interval in those with (*p* = 0.48) or without (*p* = 0.048) liver cirrhosis. In patients without liver cirrhosis, right ventricular global longitudinal strain (RV GLS) decreased from 22 (−30 to −17) to −21 (−27–18), *p* = 0.024. In patients with liver cirrhosis, lateral mitral E’ velocity was reduced from 14.38 ± 3.59 to 13.62 ± 3.21 cm/s, *p* = 0.02 and indexed left atrial volume (LAVI) was increased from 25.96 ± 3.96 to 26.86 ± 4.12 ml/m^2^, *p* = 0.032. There were no changes in both groups regarding left ventricular (LV) dimensions, ejection fraction, trans-mitral E/A ratio, E/E’ ratio, deceleration time, right ventricular (RV) systolic pressure, mean pulmonary artery pressure, RV fractional area change, tricuspid annular plane systolic excursion, and LV GLS.

**Conclusion:**

The current national protocol of HCV infection treatment with direct-acting antiviral agents used in Egyptian patients has a good cardiac safety profile. Such treatments have no effect on QTc interval, left and right ventricular functions except for a decrease in RV GLS in those with no liver cirrhosis and a reduction in lateral mitral E’ velocity in those with liver cirrhosis both remained within the normal reference range.

## Background

Hepatitis C virus (HCV) infection is a major cause of chronic liver disease worldwide. The long-term impact of HCV infection varies from minimal histological changes to extensive fibrosis and cirrhosis with or without hepatocellular carcinoma [1].

Although HCV is primarily a hepatotropic virus, it may lead to extra-hepatic manifestations. HCV was isolated from the myocardium of patients suffering from myocarditis and cardiomyopathy and is therefore additionally considered a cardiotropic virus [2].

It has been shown that up to 50% of patients with advanced liver cirrhosis may have cardiac dysfunction, a condition often called cirrhotic cardiomyopathy. Such patients have normal or increased cardiac output at rest with a blunted response to stress. They may also have QT interval prolongation and chronotropic incompetence [3].

Treatment of chronic HCV infection evolved from the limited efficacy interferon-based therapy to the highly effective direct-acting antiviral (DAA) agents. Novel DDAs have received breakthrough therapy status by the Food and Drug Administration; however, retrospective studies, case reports, and post-marketing reports suggest possible deleterious effects to the heart including bradyarrhythmia and toxic cardiomyopathy [4, 5].

Advances in trans-thoracic echocardiography with the application of strain analysis and speckle tracking provide sensitive assessment of cardiac function that may predict clinical outcomes in various heart diseases, including those with HCV infection [6, 7].

The aim of this study was to assess the effect of treatment of chronic HCV infection with DAAs on corrected QT interval (QTc) of the surface ECG and on cardiac function using trans-thoracic echocardiography.

## Methods

The study protocol received approval from the institutional ethical committee. Written informed consents were provided by all participants. A prospective cohort study was conducted on 100 treatment-naïve adult patients (≥ 18 years old) suffering from chronic HCV infection—confirmed by a polymerase chain reaction test for HCV (genotype 4). Patients were randomly selected from the institution’s specialized clinic for HCV-infected patients in the period from March 2018 to September 2019.

Patients were excluded from the study if they had a history of arrhythmias or a current arrhythmia; any type of cardiomyopathy; coronary heart diseases; valvular heart disease; uncontrolled hypertension; previously treated chronic HCV patients; concomitant infection with either of hepatitis B virus, human immunodeficiency virus or bilharziasis; patients with an estimated glomerular filtration rate less than 30 ml/min; pregnant women; those with any form of autoimmune diseases; those with a history of malignancy; and those receiving medications known to be cardiotoxic, have a negative chronotropic or antiarrhythmic effect.

Patients were assigned into two equal groups (50 each) depending on the presence of liver cirrhosis (defined as Child-Pugh score A and B) [8]. All patients were treated according to the national protocol for the management of chronic HCV infection [9, 10] as follows: those without liver cirrhosis received a combination of sofosbuvir 400 mg daily and daclatasvir 60 mg daily for 12 weeks. Those with liver cirrhosis received a combination of sofosbuvir 400 mg daily, daclatasvir 60 mg daily and ribavirin 600 mg daily for 12 weeks.

### Clinical history and baseline investigations

Detailed patient history was taken specially to define Child-Pugh score and to assess for the presence of exclusion criteria. Height in centimeters and weight in kilograms were measured to estimate body surface area (BSA) in m^2^. BSA was calculated using the Mosteller formula where BSA = square root of ([height in cm × weight in kg]/3600) [11].

Venous blood samples were obtained from all patients to measure serum albumin, total bilirubin, prothrombin time, partial thromboplastin time, international normalized ratio, liver enzymes, rapid ELISA testing of HCV antibody and hepatitis B surface antigen. Additionally, quantitative PCR of HCV antibody was assessed before the start of treatment, at 4 weeks, at the end of treatment, and 12 weeks after the end of treatment. Abdominal ultrasonography was performed at baseline to examine for criteria of liver cirrhosis, as well as, Fibroscan assessment of liver stiffness/scaring.

### Cardiac investigations

The following was performed at baseline and 1 week after the end of treatment:

12-lead surface ECG: to assess heart rate, presence of any degree of heart block, and to assess corrected QT interval.

Standard trans-thoracic echocardiography: standard trans-thoracic echocardiography with machine-integrated ECG recording was performed for all patients using an S7 machine with an M4S matrix sector array probe with a frequency range from 1.7 to 342 MHz (GE Vingmed, Horten, Norway). A standard study following was performed for all patients by an echocardiographer accredited by the European Association of Cardiovascular Imaging (EACVI) following guideline recommended protocols [12] to obtain the following measurements: Left ventricular end-diastolic diameter (LVEDD) and left ventricular end-systolic diameter (LVESD) measured by M-mode from the parasternal short-axis view at the level of the papillary muscles; left ventricular end-diastolic volume (LVEDV), end-systolic volume (LESV), and ejection fraction (LVEF) measured by the modified Simpson’s method [12]; LV volumes were then indexed according to BSA to calculate indexed LVEDV (LVEDVI) and indexed LVESV (LVESVI). Indexed left atrial volume (LAVI) was measured using the biplane Simpson’s method from the apical 4- and 2-chamber views [12].

LV diastolic function was assessed by estimating trans-mitral E/A ratio, deceleration time (DT), lateral mitral annulus tissue Doppler first negative wave velocity (E’), and E/E’ ratio.

Right ventricular (RV) function was assessed by estimating mean pulmonary artery pressure (PAP), RV systolic pressure (RVSP), tricuspid annular plane systolic excursion (TAPSE), and RV fractional area change (RVFAC). The latter was calculated as (RV end-diastolic area–RV end-systolic area)/RV end-diastolic area × 100 [13].

2-D left ventricular strain: analysis of digital loops acquired from the apical 2-, 3-, and 4-chamber views was performed using machine-integrated software for speckle tracking to calculate the peak global longitudinal strain (GLS) [14].

2-D right ventricular strain: analysis of an RV-focused apical 4-chamber view digital loop was performed using speckle tracking for off-line semi-automated analysis of the speckle-based RV free wall longitudinal strain [15].

### Statistics

Data were statistically analyzed using IBM SPSS Statistics version 25 (IBM corporation, Armonk, NY, U.S.). Categorical variables were expressed as number and percentage and analyzed using the chi-square test. Continuous variables were expressed as mean ± SD and analyzed using Student’s *t* test for variables that passed normality tests and Mann-Whitney *U* test for those that did not pass normality. Correlations were analyzed using Pearson’s correlation coefficient (*r*). A probability value *p* < 0.05 was considered statistically significant and a *p* value < 0.0001 was considered highly significant.

## Results

### Baseline characteristics

Baseline demographic and clinical characteristics, as well as assessment of liver status by abdominal ultrasonography and fibroscan, are shown in Table 1. Patients in the liver cirrhosis group were older 52 ± 12.04 versus 46.76 ± 12.1 years, *p* = 0.032. Additionally, there was a higher percentage of diabetics in the liver cirrhosis group 14 (28%) versus 7 (14%), *p* = 0.086.

**Table 1:** Comparing demographic and clinical characteristics

Variable	No cirrhosis (*n* = 50)	Cirrhosis (*n* = 50)	*p* value
Clinical characteristics			
Age, years	46.76 ± 12.1	52 ± 12.04	0.032
Male gender, *n* (%)	19 (38%)	21 (42%)	0.683
Current smoker, *n* (%)	11 (22%)	11 (22%)	1
Hypertension, *n* (%)	13 (26%)	28 (56%)	0.002
Diabetes, *n* (%)	7 (14%)	14 (28%)	0.086
Liver assessment by abdominal ultrasound at baseline			
Normal	40 (80%)	0 (0%)	< 0.0001
Parenchymatous liver disease	10 (20%)	38 (76%)	
Cirrhotic liver disease	0 (0%)	12 (24%)	
Fibroscan assessment at baseline			
F0	13 (26%)	0 (0%)	< 0.0001
F1	27 (54%)	0 (0%)	
F2	9 (18%)	0 (0%)	
F3	1 (2%)	16 (32%)	
F4	0 (0%)	34 (68%)	

Continuous variables are expressed as mean and standard deviation whereas categorical variables are expressed as number (percentage)

### Effect of treatment on cardiac function in patients with no liver cirrhosis

Treatment with direct-acting antivirals in patients with no liver cirrhosis caused a significant reduction in RV GLS from 22 (−30 to −17) to −21 (−27–18), *p* = 0.024. However, treatment had no effect on QTc interval, LV systolic and diastolic functions, and no effect on other RV functions as shown in Table 2.

**Table 2:** Comparing ECG and echocardiographic variables in patients with no liver cirrhosis before and after treatment

Variable	Before treatment (*n* = 50)	After treatment (*n* = 50)	*p* value
QTc, ms	406.64 ± 18.73	408.76 ± 14.12	0.48
LVEDD, mm	47.36 ± 3.29	47.84 ± 2.57	0.26
LVESD, mm	29.76 ± 2.81	29.6 ± 1.96	0.701
LVEDV, ml	101.08 ± 19.01	103.6 ± 17.15	0.183
LVESV, ml	54.18 ± 9.40	55.48 ± 8.69	0.205
LVEDVI, ml/m^2^	34.48 ± 7.19	33.24 ± 6.21	0.133
LVESVI, ml/m^2^	18.43 ± 3.79	17.88 ± 3.08	0.224
LVEF, %	57.3 ± 3.71	57.54 ± 4.15	0.268
LAVI, ml/m^2^	25.38 ± 4.69	25.98 ± 4.13	0.174
Trans-mitral E/A ratio	1.21 ± 0.38	1.18 ± 0.32	0.323
DT, ms	200.08 ± 31.09	204.84 ± 30.49	0.285
Lateral mitral E’, cm/s	15.2 ± 4.52	15.7 ± 3.47	0.250
E/E’ ratio	6.32 ± 2.31	6.3 ± 1.54	0.373
RVSP, mmHg	25.52 ± 5.84	24.96 ± 5.29	0.474
Mean PAP, mmHg	15.28 ± 3.68	15.28 ± 3.69	1
TAPSE, mm	24.06 ± 2.65	24.86 ± 2.94	0.052
RVFAC, %	45.64 ± 4.89	46.46 ± 5.13	0.120
LV GLS	−20 (−28 to −17)	− 20 (−24 to − 17)	0.161
RV GLS	−22 (−30 to − 17)	−21 (−27 to −18)	0.024

Continuous variables for variables that passed normality tests are expressed as mean and standard deviation while for those that did not pass normality test as median (range).QTc indicated corrected QT interval, LVEDD indicates left ventricular end-diastolic dimension, LVESD indicates left ventricular end-systolic dimension, LVEDV indicates left ventricular end-diastolic volume, LVESV indicates left ventricular end-systolic volume, LVEDVI indicates indexed left ventricular end-diastolic volume, indexed LVESVI indicates left ventricular end-systolic volume, LVEF indicates left ventricular ejection fraction, LAVI indicated indexed left atrial volume, RVSP indicated right ventricular systolic pressure, PAP indicated pulmonary artery pressure, TAPSE indicates tricuspid annular plane systolic excursion, RVFAC indicates right ventricular fractional area change, LV GLS indicates left ventricular global longitudinal strain, RV GLS indicates right ventricular global longitudinal strain

### Effect of treatment on cardiac function in patients with liver cirrhosis

Child-Pugh score’s distribution in patients with liver cirrhosis was A5 = 31 (62%), A6 = 7 (14%), and B7 = 12 (24%) patients.

Treatment with direct-acting antivirals in patients with liver cirrhosis caused a reduction in lateral mitral E’ from 14.38 ± 3.59 to 13.62 ± 3.21 cm/s, *p* = 0.02 and an increase in LAVI from 25.96 ± 3.96 to 26.86 ± 4.12 ml/m^2^, *p* = 0.032. However, treatment had no effect on QTc interval, other measures of LV systolic and diastolic functions, and RV functions as shown in Table 3.

**Table 3:** Comparing ECG and echocardiographic variables in patients with liver cirrhosis before and after treatment

Variable	Before treatment (*n* = 50)	After treatment (*n* = 50)	*p* value
QTc, ms	415.84 ± 24.40	410.96 ± 29.41	0.48
LVEDD, mm	48.14 ± 2.81	49.3 ± 2.33	0.26
LVESD, mm	29.8 ± 2.44	30.18 ± 2.04	0.701
LVEDV, ml	94.96 ± 18.67	96.84 ± 17.71	0.183
LVESV, ml	31.16 ± 6.51	31.92 ± 5.32	0.359
LVEDVI, ml/m^2^	52.98 ± 9.08	54.14 ± 9.59	0.133
LVESVI, ml/m^2^	17.4 ± 3.01	17.82 ± 2.8	0.354
LVEF, %	66.04 ± 2.98	65.42 ± 4.9	0.41
LAVI, ml/m^2^	25.96 ± 3.96	26.86 ± 4.12	0.032
Trans-mitral E/A ratio	1.09 ± 0.38	1.1 ± 0.36	0.678
DT, ms	182.36 ± 30.85	180.98 ± 21.85	0.751
Lateral mitral E’, cm/s	14.38 ± 3.59	13.62 ± 3.21	0.02
E/E’ ratio	6.77 ± 2.44	6.61 ± 2.22	0.62
RVSP, mmHg	26.34 ± 6.06	27.54 ± 7.15	0.131
Mean PAP, mmHg	16 ± 3.1	16.86 ± 3.47	0.097
TAPSE, mm	24.56 ± 3.08	23.94 ± 2.8	0.164
RVFAC, %	45.72 ± 4.88	44.98 ± 4.86	0.149
LV GLS	−20 (−26 – −16.5)	− 20 (−24 – − 16)	0.198
RV GLS	−22 (−30 – −17)	−21 (− 30 – − 16)	0.122

Continuous variables for variables that passed normality tests are expressed as mean and standard deviation while for those that did not pass normality test as median (range).QTc indicated corrected QT interval, LVEDD indicates left ventricular end-diastolic dimension, LVESD indicates left ventricular end-systolic dimension, LVEDV indicates left ventricular end-diastolic volume, LVESV indicates left ventricular end-systolic volume, LVEDVI indicates indexed left ventricular end-diastolic volume, indexed LVESVI indicates left ventricular end-systolic volume, LVEF indicates left ventricular ejection fraction, LAVI indicated indexed left atrial volume, RVSP indicated right ventricular systolic pressure, PAP indicated pulmonary artery pressure, TAPSE indicates tricuspid annular plane systolic excursion, RVFAC indicates right ventricular fractional area change, LV GLS indicates left ventricular global longitudinal strain, RV GLS indicates right ventricular global longitudinal strain

## Discussion

HCV infection is a major cause of chronic liver diseases and liver cirrhosis worldwide. It is associated with numerous hepatic and extra-hepatic manifestations including cardiac affection [1, 2]. Novel treatment regimens in the form of direct-acting antivirals that target different steps in the HCV lifecycle have proven to be effective. However, their cardiac safety remains in question [5].

This study aimed to assess the effect of treatment of chronic HCV infection with DAAs used in the national protocol for the management of chronic HCV infection on QTc interval of the surface ECG and on cardiac function using trans-thoracic echocardiography. All patients were treatment-naïve with 50 patients without liver cirrhosis and 50 patients with liver cirrhosis (Child-Pugh score A and B).

The main findings of this study are that DAAs used in the national Egyptian treatment protocol for HCV infection treatment have no effect on QTc interval in both patients with and without liver cirrhosis. RV GLS decreased in those with no liver cirrhosis after treatment while lateral mitral E’ velocity was reduced and LAVI was increased in those with liver cirrhosis after treatment with all measurements remaining within the normal reference range. Additionally, these agents have no effect on left and right ventricular dimensions, volumes, other systolic, and diastolic functions as assessed by the various guideline-recommended measurements [12–15]. To the best of our knowledge, this is the first study to assess RV GLS in HCV patients treated with DAAs.

Baseline patient characteristics showed patients in the group with cirrhosis to be older which is expected as cirrhosis is part of disease progression that occurs over time. Additionally, hypertension was more common in those with liver cirrhosis. This could be attributed to advanced age and the pathological changes that occur with progressive liver cirrhosis. A study by Novo et al. in 2018 on 39 patients with HCV-induced liver cirrhosis without previous cardiovascular events and 39 controls without liver or cardiovascular disease examined the early features of cardiovascular damage in HCV-compensated cirrhotic patients treated with DAAs using myocardial deformation indices. In this study, both diabetes mellitus and hypertension were more common among cirrhotic patients [16].

As mentioned, QTc interval did not change after treatment in this study. This was investigated by others. In 2017, Biomy et al. investigated the cardiovascular effects of DAAs in 170 patients with HCV infection. They divided their patients into two groups: group 1 (*n* = 100) received pegylated interferon alfa, sofosbuvir, and ribavirin while group 2 (*n* = 70) received sofosbuvir and simeprevir. After 6 to 12 months of follow-up, their results showed that there was no change in the QTc interval. Additionally, no arrhythmias were observed throughout the study and during follow-up visits [17]. Also, in 2017 Durante-Mangoni et al. examined the ECG of 39 HCV infection patients treated with either a sofosbuvir- (*n* = 26) or a non-sofosbuvir-based treatment (*n* = 13). ECG tracings were obtained on the first day of treatment then after 7, 14, and 28 days. Their results showed that for the sofosbuvir group QTc interval significantly increased at 1 week (*p* = 0.013) then returned to baseline values later during therapy till the end of treatment [18].

Minor changes occurred in a couple of measurements of diastolic function in patients from the current study who had liver cirrhosis with lateral mitral E` being reduced and LAVI increased. All other diastolic parameters did not change after treatment. In the study by Novo et al. lateral E’ velocity decreased after treatment (*p* = 0.001) [16]. However, in that by Biomy et al., no changes occurred in E/A ratio, DT, and E/E` [17].

The current study showed no changes in LV volumes and EF after treatment in both patients with and without liver cirrhosis. This was evaluated in 2018 in a study by El-Adawy et al. that examined the effect of different HCV infection treatment regimens on the heart using cardiac magnetic resonance imaging (CMR) and creatine kinase MB fraction (CK-MB) in 390 patients. Patients were divided into four groups according to the treatment regimen. Group A was treated with ledipasvir and sofosbuvir (LED + SOF), group B was treated with simepriver and sofosbuvir (SIM + SOF), group C was treated with sofosbuvir and daclatasvir (SOF + DCV), and group D was treated with sofosbuvir, daclatasvir, and ribavirin (SOF + DCV + RBV). CMR was done before starting treatment, during follow-up and after 6 months of treatment. Groups C and D showed a significant elevation of CK-MB levels with increased edema, early and late gadolinium enhancement compared with group B. These changes were fully reversible after the stoppage of treatment without leaving any permanent cardiac damage [19]. In concordance with the current study, the studies by Novo et al. [16] and Biomy et al. [17] showed no differences in LV dimensions, volumes, and EF before and after treatment.

RV echocardiographic parameters such as TAPSE, RVFAC, RVSP, and mean PAP showed no change after treatment. Biomy et al. had similar findings [17] while Novo et al. agreed except for TAPSE which was markedly reduced after treatment in their study (*p* ˂ 0.01) [16].

Regarding GLS assessment of the left and right ventricles in the current study, only RV GLS showed a significant reduction after treatment in patients without liver cirrhosis. A study in 2018 by Mazzitelli et al. measured possible changes in cardiovascular function in 82 patients with chronic HCV infection before and after sofosbuvir-based regimens using both LVEF and LV GLS Interestingly, LV GLS in non-cirrhotic patients improved from −20.8% at baseline to −21.4% at 1 month then worsened to −20.3% at the end of treatment (*p* = 0.031) [20]. On the other hand, Novo et al. found no changes in LV GLS after treatment [16].

We could only find one study that measured RV GLS using speckle tracking echocardiography in 49 patients with liver cirrhosis and 34 healthy controls. They concluded that RV GLS had a negative correlation to cirrhosis severity (defined as Child-Pugh score) (*r* = −0.60, *p* < 0.0001) [21]. However, no previous study examined the effect of treatment with DAAs on RV GLS.

The reduction in the RV GLS only in those without liver cirrhosis could be explained by the changes in systemic hemodynamics caused by liver cirrhosis. Patients with liver cirrhosis have a hyperdynamic circulation with increased cardiac output and heart rate, large blood volume, low total peripheral resistance, and low or normal blood pressure [22–24]. Additionally, those with liver cirrhosis received ribavirin 600 mg daily in addition to the DAAs used in both groups in accordance with the national protocol of management of HCV infection. Ribavirin is known to cause significant anemia which in turn has an effect on systemic hemodynamics [25]. Such different hemodynamics between those with and without liver cirrhosis could explain the difference.

### Study limitations

Limitations of the current study are that it comes from a single medical center, follow-up was performed only once 1 week after the end of treatment, patients with underlying cardiac conditions were excluded. The use of ribavirin in the patients with liver cirrhosis may be considered a confounding factor; however, we had to abide by the national treatment protocols.

## Conclusion

The current national protocol of HCV infection treatment with direct-acting antiviral agents used in Egyptian patients has a good cardiac safety profile. Such treatments have no effect on QTc interval, left and right ventricular functions except for a decrease in RV GLS in those with no liver cirrhosis and a reduction in lateral mitral E’ velocity in those with liver cirrhosis both remained within the normal reference range.

## Data Availability

The datasets used and analyzed during the current study are available from the corresponding author on reasonable request.

## Abbreviations

BSA: Body surface area
DAA: Direct-acting antivirals
DT: Deceleration time
EACVI: European Association of Cardiovascular Imaging
ECC: Electrocardiogram
ELISA: Enzyme linked immunosorbent assay
FAC: Fractional area change
GE: General Electric
GLS: Global longitudinal strain
HCV: Hepatitis C virus
LAVI: Indexed left atrial volume
LV: Left ventricle
LVEDD: Left ventricular end-diastolic diameter
LVEDV: Left ventricular end-diastolic volume
LVEDVI: Indexed left ventricular end-diastolic volume
LVEF: Left ventricular ejection fraction
LVESD: Left ventricular end-systolic diameter
LVESV: Left ventricular end-systolic volume
LVESVI: Indexed left ventricular end-systolic volume
MHz: Megahertz
PAP: Pulmonary artery pressure
PCR: Polymerase chain reaction
QTc: Corrected QT interval
RV: Right ventricle
RVSP: Right ventricular systolic pressure
TAPSE: Tricuspid annular plane systolic excursion
